# Breaking through Electrospinning Limitations: Liquid-Assisted Ultrahigh-Speed Production of Polyacrylonitrile Nanofibers

**DOI:** 10.1021/acsaenm.4c00657

**Published:** 2024-12-03

**Authors:** John Schossig, Qiangjun Hao, Tyler Davide, Adedayo Towolawi, Cheng Zhang, Ping Lu

**Affiliations:** † Department of Chemistry and Biochemistry, Rowan University, Glassboro, New Jersey 08028, United States; ‡ Chemistry Department, Long Island University (Post), Brookville, New York 11548, United States

**Keywords:** liquid-assisted ultrahigh-speed electrospinning (LAUHS-ES), taylor cone stabilization, polyacrylonitrile (PAN) nanofibers, carbon-based nanofibers, high-throughput nanofiber production

## Abstract

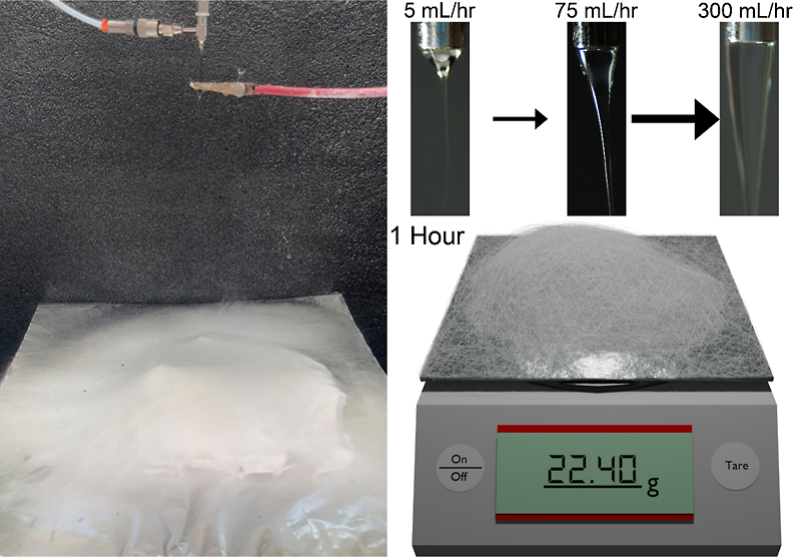

Carbon-based nanofibers are critical materials with broad applications in industries such as energy, filtration, and biomedical devices. Polyacrylonitrile (PAN) is a primary precursor for carbon nanofibers, but conventional electrospinning techniques typically operate at low production rates of 0.1–1 mL/h from a single spinneret, limiting scalability. In this study, we introduce a novel liquid-assisted ultrahigh-speed electrospinning (LAUHS-ES) technique that achieved actual production rates over 220 times faster than conventional methods. This dramatic increase in throughput is achieved through Taylor cone stabilization using a thin layer of liquid sheath, allowing for ultrahigh-speed electrospinning without compromising the structural integrity or uniformity of the nanofibers. Comprehensive characterization, including scanning electron microscopy (SEM), atomic force microscopy (AFM), Fourier-transform infrared spectroscopy (FTIR), and X-ray diffraction (XRD), confirmed the high quality, consistency, and crystallinity of the produced nanofibers. Our results demonstrate that PAN nanofiber fabrication can be scaled up significantly while maintaining precise control over fiber morphology and performance. This advancement holds substantial promise for large-scale industrial applications, enabling more efficient and cost-effective production of carbon-based nanofibers.

## Introduction

Nanofibers have garnered significant attention in recent years due to their unique properties and wide-ranging applications across various industries.^1−3^ Their tunable features, including size, composition, porosity, surface area-to-volume ratio, wettability, and mechanical and electrical characteristics, make them highly versatile for applications such as drug delivery systems, catalyst substrates, energy storage, tissue engineering, and air filtration systems.^4−17^ Among the different fabrication methods, electrospinning has emerged as a highly effective and scalable technique for producing nanofibers from a wide variety of polymers.^18−20^ It offers simplicity, cost-efficiency, and the flexibility to produce nanofibers with customizable properties.^21−23^ However, the major limitation of traditional electrospinning techniques lies in the extremely slow production rates from a single spinneret, typically limited to 0.1–2 mL/h, due to challenges such as maintaining the stability of the Taylor cone, solvent evaporation, and precise control over fiber morphology.^24−26^ These factors restrict the throughput of high-quality nanofibers to milligram level per hour or less from a single spinneret.

Polyacrylonitrile (PAN) is a cost-effective, easily processable homopolymer widely used in the production of nanofibers due to its exceptional mechanical strength, chemical resistance, and thermal stability.^27−29^ It is particularly valued for its high surface area-to-volume ratio, which makes it suitable for applications where enhanced surface interaction is critical, such as in filtration, catalysis, and energy storage.^30,31^ PAN nanofibers are also highly tailorable, allowing for modification of their mechanical, chemical, and physical properties to suit a variety of applications.^28^ Despite its versatility, scaling up PAN nanofiber production beyond the conventional flow rates (∼1 mL/h per spinneret) has remained a significant challenge. At higher production rates, issues such as instability of the Taylor cone, rapid solvent evaporation, and inconsistent fiber diameters arise, resulting in compromised fiber quality and batch uniformity.^32−35^ These limitations necessitate the development of novel techniques that can stabilize the electrospinning process at higher flow rates while maintaining precise control over fiber morphology and properties. PAN’s importance extends beyond its use as a nanofiber material; it is the dominant precursor for carbon nanofiber (CNF) production, accounting for more than 90% of global CNF manufacturing.^36^ During carbonization, PAN undergoes cyclization, dehydrogenation, and graphitization processes that transform it into high-performance carbon fibers with excellent electrical conductivity, thermal stability, and tensile strength.^37−39^ These properties make PAN-derived carbon nanofibers indispensable in advanced applications such as aerospace, automotive, energy storage, and composite materials.^40−42^ As demand for high-performance carbon nanofibers continues to grow, there is a pressing need to overcome the production bottlenecks associated with PAN electrospinning to enable large-scale, high-throughput manufacturing of this critical precursor material.

Efforts to overcome the production limitations in electrospinning have led to various approaches, but many have fallen short of significantly increasing nanofiber throughput without compromising fiber quality. One widely used method involves blending PAN with a second polymer solution before electrospinning (blend electrospinning) or utilizing a biphasic needle to produce Janus or conjugate fibers (conjugate electrospinning).^43−46^ While these techniques allow researchers to leverage PAN’s mechanical strength and ability to produce fine-diameter fibers, they often introduce complications in fiber morphology, such as uneven surface textures or enlarged diameters.^47^ Additionally, these methods are limited in their ability to produce more complex fiber architectures, such as those created via coaxial electrospinning, which allows for core–shell structures and encapsulation, critical for applications such as drug delivery or advanced composite materials.^48^ Other approaches to increase production rates include needleless electrospinning, where the polymer solution is ejected from a free surface rather than through a needle.^49,50^ While this technique can produce larger quantities of fibers, it lacks the precision and control required to maintain the quality and uniformity needed for advanced applications.^50^ Further, large solvent evaporation is usually associated with needleless electrospinning, making it environmentally unfriendly and economically unfeasible for large-scale operations.^51^ Similarly, multineedle electrospinning setups have been explored to boost fiber output by employing several spinnerets simultaneously. However, this method introduces new challenges, such as uneven electric field distribution and complex interactions between the needles, which can compromise fiber uniformity and require sophisticated instrumentation to mitigate.^52,53^

Conventional liquid-assisted electrospinning, in which a solvent sheath is applied around the polymer solution, has shown promise in improving the electrospinnability of polymers like PAN by reducing needle clogging and inconsistent jet formation.^54^ While this technique improves the process stability, it does not significantly enhance nanofiber production rates due to limitations in the flow rate that the sheath liquid can support.^55^ Even though liquid-assisted methods stabilize the process and enhance fiber quality, their ability to scale production to an industrially relevant level has remained limited.^56^ This underscores the need for innovative techniques that not only improve the electrospinning process but also achieve high-speed production without sacrificing fiber properties.^57^ Our novel liquid-assisted ultrahigh-speed electrospinning (LAUHS-ES) method effectively addresses the longstanding challenges of low production rates in conventional electrospinning by enabling substantially higher throughput without sacrificing fiber quality. This technique utilizes a coaxial electrospinning setup in which PAN serves as the core solution, while a carefully selected liquid sheath surrounds the core, stabilizing the Taylor cone and allowing for ultrahigh-speed production. The solvent sheath acts as a protective barrier, shielding the polymer jet from ambient conditions and preventing deformation of the nanofibers, even at elevated flow rates. To optimize the process, three solvents—polar aprotic tetrahydrofuran (THF), nonpolar chloroform (CHCl_3_), and nonpolar diethyl ether—were investigated based on their polarity and solubility with PAN. Each solvent was tested across a range of flow rates to identify the optimal combinations for stable, high-throughput fiber production. This innovative approach achieves production speeds several orders of magnitude greater than conventional techniques, overcoming the limitations associated with inconsistent fiber diameters, low flow rates, and instability. By dramatically increasing throughput while maintaining precise control over fiber morphology, the LAUHS-ES technique paves the way for large-scale, cost-effective manufacturing of high-quality nanofibers, meeting industrial demands for materials such as carbon fiber precursors.

## Experimental Section

### Chemicals and Materials

Polyacrylonitrile (PAN) polymer solutions were prepared by dissolving 10% wt./wt. PAN (Sigma-Aldrich, molecular weight 150,000 Da, (C_3_H_3_N)_*n*_) in dimethylformamide (DMF, ≥99.9%, BTC). The solutions were stirred at 120 rpm for 24 h at room temperature (approximately 25 °C) to ensure complete dissolution and homogeneity prior to electrospinning. Three different solvents were used as the liquid sheaths in the coaxial electrospinning process: tetrahydrofuran (THF, ≥99.5%, TCI America), diethyl ether (ACS grade, Pharmco), and chloroform (CHCl_3_, ≥99.5%, TCI America). Both THF and chloroform were stored at room temperature, while diethyl ether was maintained at 10 °C to minimize evaporation due to its high volatility.

### LAUHS-ES of PAN

Nanofiber electrospinning was conducted using two KD Scientific Legato Series 110 Programmable Syringe Pumps to control the flow of both the polymer core solution and the solvent sheath. A high voltage power supply (ES30P-10W Gamma High Voltage Research) provided the required electric field for the electrospinning process. All electrospinning experiments were performed at room temperature (∼23 °C) with a relative humidity of ∼40%. The PAN polymer solution was loaded into a 5 mL BD syringe, connected to the core (22G input) of a coaxial needle. This syringe was mounted on one of the syringe pumps, which controlled the flow rate of the polymer solution. The solvent sheath solution was loaded into a separate 5 mL glass Cadence Science syringe, connected to the sheath (18G input) of the coaxial needle via PTFE Teflon tubing, and mounted on the second syringe pump. The electrospinning setup was positioned above a 30 × 30 cm aluminum foil collection surface, which was placed on an adjustable jack stand 20 cm below the coaxial needle tip. The applied voltage varied depending on the solvent used for the sheath solution. For electrospinning using chloroform, a voltage of 15.0 kV was applied, whereas a voltage of 20.0 kV was used for experiments involving THF and diethyl ether. Nanofiber samples were collected for 1–3 min, with varying flow rates used for different trials to test the LAUHS-ES technique.

### Characterization

The surface morphology of the electrospun nanofibers was analyzed using scanning electron microscopy (SEM, Apreo S, FEI). Prior to imaging, all samples were sputter-coated with a thin layer of gold for 30 s to enhance conductivity. SEM imaging was conducted at a working distance of 6 mm, using an accelerating voltage of 10 kV and a current of 0.4 nA. Fiber diameters were measured using ImageJ software (version 1.53t). Cross-sectional SEM images were obtained by first freezing the nanofibers in liquid nitrogen, followed by breaking them laterally to expose the internal structure. The electrospinning process was recorded in real-time using a digital camera (EOS R1, Canon) equipped with a macro lens (EF 100 mm f/2.8 L Macro IS USM, Canon) to capture dynamic changes in the Taylor cone. Still images were extracted from these recordings to analyze cone stability and deformation under different processing conditions. Atomic force microscopy (AFM, Bruker Dimension XR) was also employed to examine the surface structure of the PAN nanofibers in finer detail. The nanofibers were electrospun directly onto mica substrates (highest grade V1 mica discs, 12 mm, Electron Microscopy Sciences). AFM imaging was performed in tapping mode using an AppNano ACT silicon probe (tip radius <10 nm, resonant frequency = 300 kHz, spring constant = 37 N/m) at a scan rate of 0.25 Hz. The AFM data were processed and rendered into 3D surface models using Nanoscope Analysis 3.0 software. The chemical composition of the nanofibers was investigated using attenuated total reflectance Fourier transform infrared spectroscopy (ATR-FTIR, PerkinElmer Frontier). Spectra were recorded over a range of 4000–650 cm^–1^, with a resolution of 4 cm^–1^, and averaged over 64 scans to ensure accurate spectral data. X-ray diffraction (XRD, Bruker D8 Discover) was employed to assess the crystallinity of the nanofibers. XRD measurements were performed using Cu Kα radiation, scanning from 2θ = 5° to 90°, with a step size of 0.02° and a count time of 0.5 s per step. The instrument was operated at 40 kV and 40 mA, ensuring optimal resolution for the identification of crystalline phases.

## Results and Discussion

### LAUHS-ES of PAN

**Figure 1:**
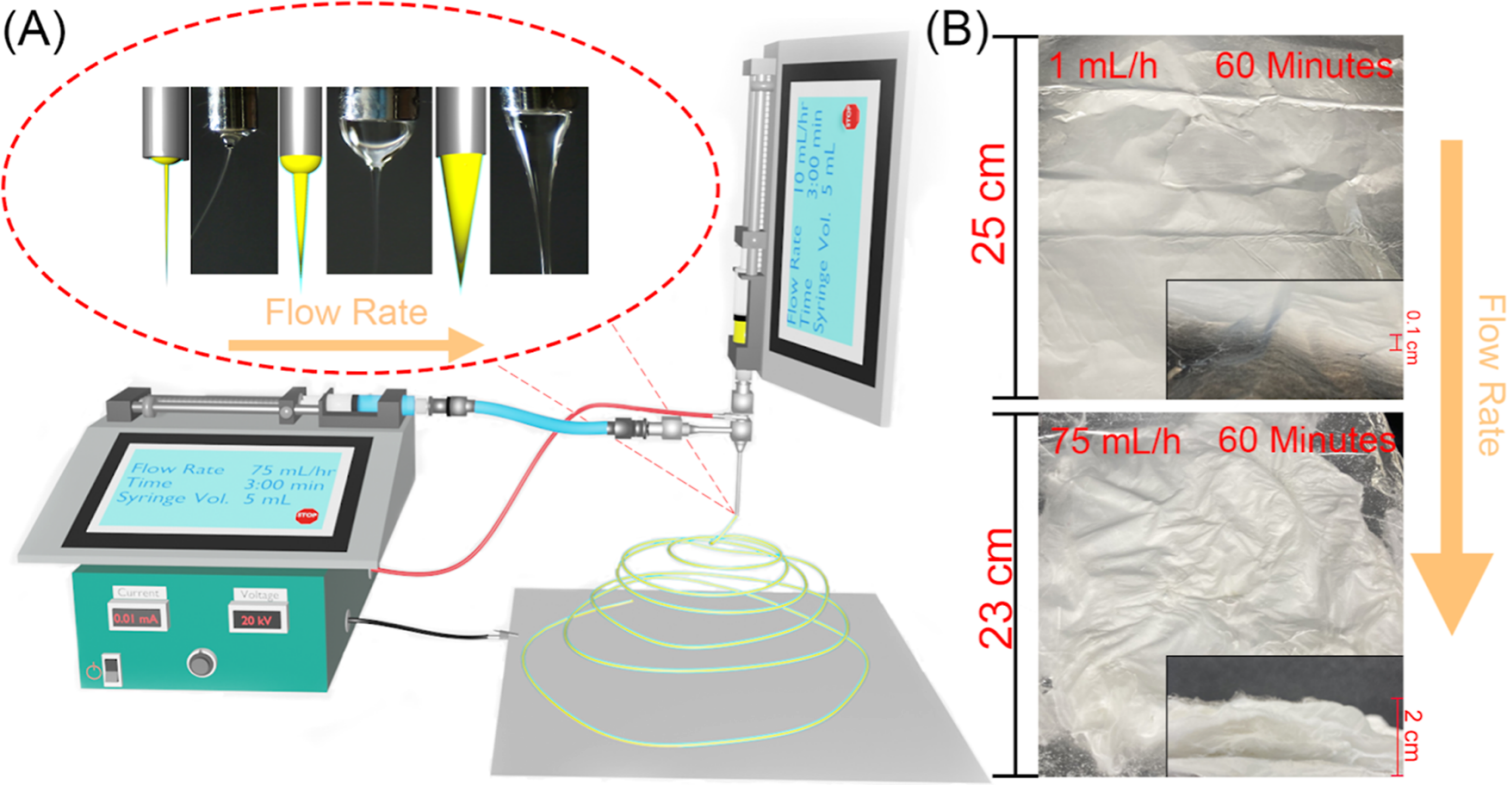
(A) Schematic representation of the LAUHS-ES setup, highlighting the gradual change in Taylor cone geometry as the flow rate increases. Inset shows the variation in Taylor cone shape from initial jet formation to stable cone. (B) Fiber membranes produced after 1 h of electrospinning using conventional electrospinning at 1 mL/h (top) and the novel LAUHS-ES technique at 75 mL/h (bottom), demonstrating a significant increase in production efficiency. Insets provide a closer view of the fiber surface and membrane thickness.

The electrospinning process, as depicted in Figure 1A, demonstrates the setup used for the LAUHS-ES technique. In this configuration, the core (yellow) represents the PAN solution, while the sheath (blue) represents the solvent used to stabilize the Taylor cone. The system operates with two syringe pumps that precisely control the flow rates of both the PAN solution and the sheath liquid. This ability to fine-tune the flow rates is crucial for maintaining a stable electrospinning process, particularly at higher flow rates. As shown in the inset of Figure 1A, increasing the flow rate leads to the widening of the Taylor cone tip. Taylor cones with a larger tip were found to be more stable, promoting consistent and uniform nanofiber production. In contrast, a thinner Taylor cone tip was observed to be less stable, which negatively impacted fiber uniformity. Thus, the geometry of the Taylor cone plays a critical role in ensuring the success of the LAUHS-ES process, especially at ultrahigh flow rates. Figure 1B demonstrates the dramatic improvement in fiber production when comparing conventional electrospinning (1 mL/h) to LAUHS-ES (75 mL/h). The membrane produced at the lower flow rate (top panel) shows a significantly smaller production volume and less uniform fiber structure after 60 min of operation. In contrast, the bottom panel, representing the fiber membrane produced using LAUHS-ES, clearly shows a substantial increase in the fiber mat’s size and volume after the same duration. The inset images further reveal the enhanced thickness and homogeneity of the fibers produced using LAUHS-ES. The volume capacity of the syringe in the LAUHS-ES system was 5 mL, requiring frequent refilling to accommodate the high flow rate of 75 mL/h over the 60 min period. This could be easily optimized in scaling by increasing the syringe size, making the technique even more efficient for large-scale industrial applications. The comparison highlights the remarkable increase in production achieved with LAUHS-ES, which is up to hundreds of times higher than conventional electrospinning, all while maintaining fiber consistency and quality.

**Figure 2:**
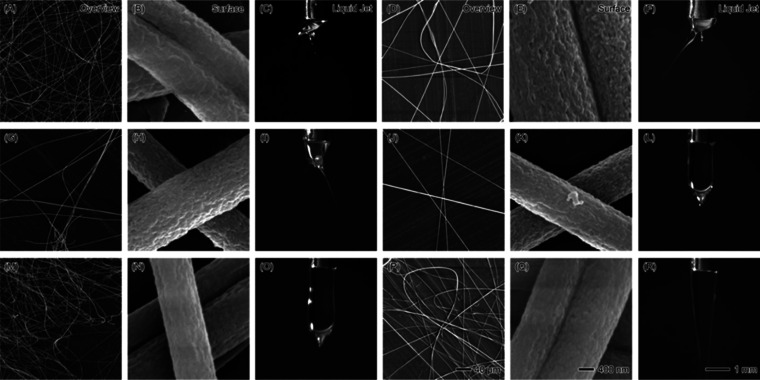
SEM images and liquid jet photographs showing the effects of increasing PAN core flow rates with a constant sheath flow rate of 1 mL/h CHCl_3_. The top row (A–C) corresponds to 1 mL/h PAN flow rate, the second row (D–F) to 5 mL/h, the third row (G–I) to 10 mL/h, the fourth row (J–L) to 20 mL/h, the fifth row (M–O) to 30 mL/h, and the last row (P–R) to 40 mL/h. Each set includes an overview of the nanofibers (left column), SEM images of fiber surface morphology (middle column), and images of the liquid jet formation (right column). Scale bars: 40 μm (overview), 400 nm (surface morphology), and 1 mm (liquid jet).

The morphology and stability of PAN nanofibers produced at different core flow rates (1–40 mL/h) with a constant sheath flow rate of 1 mL/h chloroform (CHCl_3_) were investigated using SEM and real-time liquid jet imaging, as depicted in Figure 2. The figure provides both an overview of the nanofiber mats, detailed SEM images of fiber surfaces, and snapshots of the liquid jet formation during electrospinning, offering insights into the effect of varying PAN flow rates on fiber morphology, stability, and jet formation. At a low PAN flow rate of 1 mL/h (Figure 2A–C), the fibers appear thin, uniform, and well-dispersed, consistent with the results of our previous study.^58−60^ The surface morphology (Figure 2B) shows smooth fibers, typical of PAN, but the instability of the jet (Figure 2C) indicates some fluctuations during the process. This instability arises from the relatively high ratio of chloroform to PAN (1:1), which destabilizes the jet and makes consistent fiber formation more challenging. The Taylor cone produced at this low PAN flow rate is thin and prone to oscillation due to insufficient polymer solution for continuous jet formation. As the PAN flow rate increases to 5 mL/h (Figure 2D–F), fiber density significantly increases, as observed in the overview (Figure 2D), and the fiber surfaces (Figure 2E) begin to exhibit slight roughness or porosity. This porosity can be attributed to the fast evaporation of chloroform, which allows phase separation to occur during fiber formation, leading to the development of pores. The Taylor cone at this flow rate (Figure 2F) becomes slightly more stable than at 1 mL/h, but the presence of a secondary jet indicates persistent instability, potentially leading to variations in fiber diameter or morphology. At a PAN flow rate of 10 mL/h (Figure 2G–I), the fibers become more interconnected, and the surface roughness increases (Figure 2H). The higher polymer content at this flow rate allows for more consistent fiber production, but the jet instability observed in Figure 2I continues to affect the uniformity of the fibers, as reflected in occasional thick and thin fiber segments. The increasing size of the Taylor cone suggests an unstable process at this flow rate, with intermittent dripping of polymer solution.

Further increasing the PAN flow rate to 20 mL/h (Figure 2J–L) results in thicker and more densely packed fibers (Figure 2J), but the surface of the fibers (Figure 2K) becomes increasingly porous. The chloroform’s high volatility allows for rapid solvent evaporation, which enhances phase separation and contributes to pore formation. However, the Taylor cone observed in Figure 2L is noticeably unstable, with irregularities and liquid dripping, indicating that this flow rate exceeds the stable operating range for the CHCl_3_ sheath at 1 mL/h. The instability of the jet becomes a limiting factor for uniform fiber formation, resulting in variable fiber diameters. At 30 mL/h and 40 mL/h (Figure 2M–R), the instability of the Taylor cone becomes more pronounced, as shown in Figure 2O,R. The larger volume of PAN being ejected leads to inconsistent jet formation and the dripping of the polymer solution, preventing continuous fiber production. As a result, the fiber mats (Figure 2M,P) display nonuniform thickness and uneven fiber distribution. The surface morphologies (Figure 2N,Q) further show increased porosity and roughness, emphasizing the impact of the high flow rate on fiber consistency. The liquid jets at these higher flow rates show clear signs of instability, with a swollen, irregular Taylor cone and polymer droplets forming rather than a stable fiber jet. The overall trend observed in Figure 2 suggests that while increasing the PAN flow rate results in thicker fiber mats and increased production, it also introduces significant challenges related to jet stability and fiber uniformity. The fast evaporation rate of chloroform, coupled with the high flow rates of PAN, contributes to jet instability and phase separation, resulting in porous fiber surfaces. Beyond 20 mL/h, the process becomes increasingly unstable, with large, irregular Taylor cones, multiple tips, and frequent dripping, severely affecting the quality and uniformity of the nanofibers. For stable and uniform fiber production, the PAN flow rate should be optimized to balance throughput with the stability of the electrospinning process.

**Figure 3:**
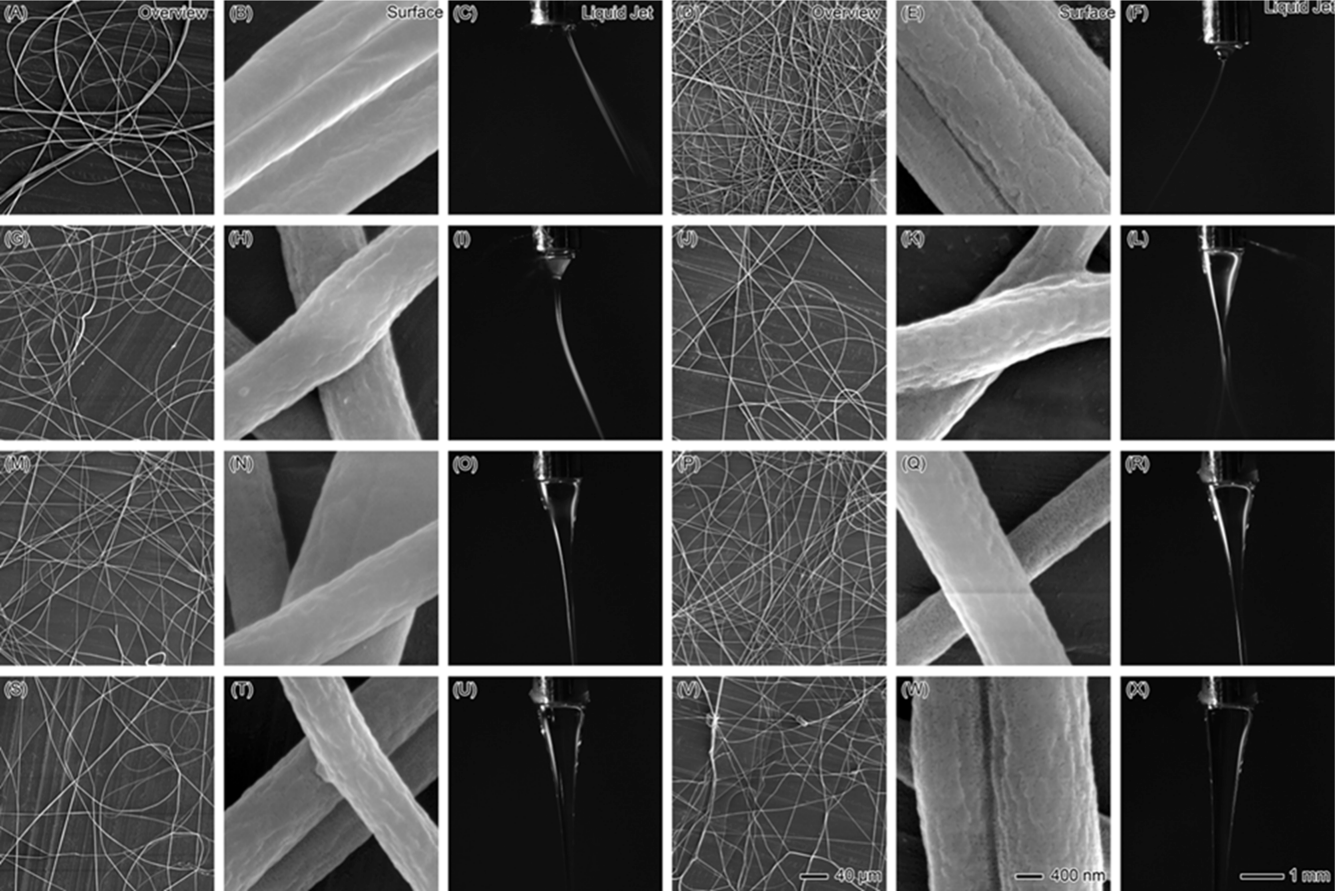
SEM images and liquid jet photographs illustrating the effects of increasing PAN core flow rates while maintaining a constant sheath flow rate of 5 mL/h ether. (A–C) 0.1 mL/h PAN, (D–F) 1 mL/h PAN, (G–I) 5 mL/h PAN, (J-L) 10 mL/h, (M–O) 20 mL/h PAN, (P–R) 30 mL/h PAN, (S–U) 40 mL/h PAN, (V–X) 50 mL/h PAN. The scale bar in (V) applies to all overviews (40 μm), the scale bar in (W) applies to all surface morphologies (400 nm), and the scale bar in (X) applies to all Liquid jets (1 mm).

The effects of varying PAN core flow rates, ranging from 0.1 to 50 mL/h, with a constant diethyl ether sheath flow rate of 5 mL/h, were examined to assess the impact on nanofiber morphology and the stability of the Taylor cone using more volatile sheath liquid during electrospinning (Figure 3). At lower PAN flow rates (0.1–30 mL/h), the electrospinning process demonstrated relative stability, producing uniform nanofibers with consistent diameters and smoother surfaces. At 0.1 mL/h PAN (Figure 3A–C), the Taylor cone was small and the tip was narrow, resulting in thin, well-distributed fibers (A) with smooth surfaces (B). The liquid jet (C) remained stable at this low flow rate. As the PAN flow rate increased to 1 mL/h (Figure 3D–F), the Taylor cone expanded slightly, but remained stable, producing denser fiber mats (D) with smooth surfaces (E). The cone tip remained narrow, and minor jet instability (F) was noted. At 5 mL/h PAN (Figure 3G–I), the fibers thickened, becoming more interconnected (G) with the surface starting to exhibit slight roughness (H). The Taylor cone (I) remained stable, but showed widening at the base. At a PAN flow rate of 10 mL/h (Figure 3J–L), fiber density increased further (J) and the surface roughness became more pronounced (K). The Taylor cone (L) continued to widen, showing early signs of instability, yet remained sufficiently stable for consistent fiber production. This trend continued at 20 mL/h PAN (Figure 3M–O), where the fibers became even denser (M) and the surface morphology displayed more significant roughness (N). The Taylor cone (O) grew larger, but remained relatively stable, although minor polymer dripping was occasionally observed. The maximum stability was achieved at 30 mL/h PAN (Figure 3P–R), where the fibers were densely packed (P), and the surface roughness was further enhanced (Q). At this point, the Taylor cone tip (R) was large and nearly occupying the entire needle tip, indicating that the cone was at its maximum stability while producing uniform nanofibers. However, when the PAN flow rate increased beyond 40 mL/h (Figure 3S–U), the Taylor cone became visibly unstable, as seen in the deformed jet (U). This instability affected the fiber morphology, resulting in wet and nonuniform fibers (S). The surface morphology (T) exhibited more pronounced porosity, likely due to the increased flow rate and solvent interaction with the polymer. At 50 mL/h PAN (Figure 3V–X), the process became highly unstable, leading to frequent polymer dripping (X) and the formation of wet fibers (V) with rough, porous surfaces (W).

**Figure 4:**
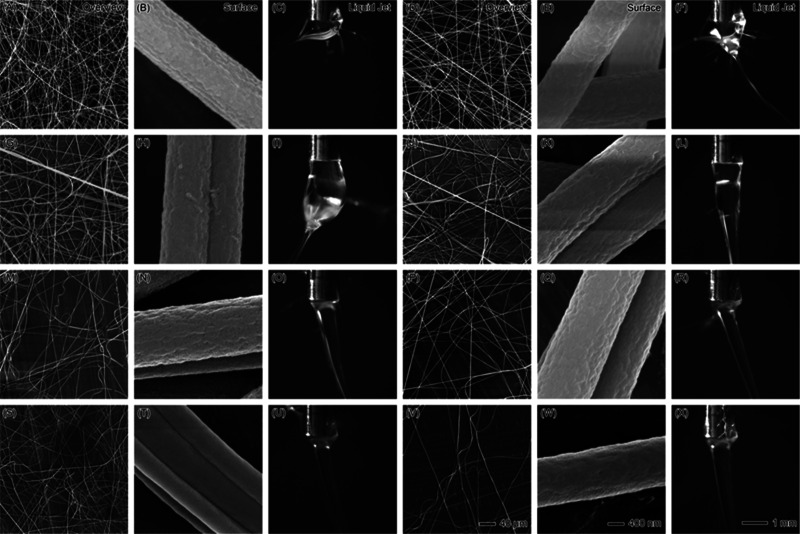
SEM images and liquid jet photographs illustrating the effects of increasing PAN core flow rates with a constant sheath flow rate of 10 mL/h tetrahydrofuran (THF). (A–C) Correspond to 1 mL/h PAN, showing sparse fiber formation in the overview (A), smooth surface morphology (B), and a stable, narrow Taylor cone in the liquid jet (C). (D–F) At 10 mL/h PAN show a denser fiber mat (D), smooth fiber surfaces (E), and a stable, slightly wider Taylor cone (F). (G–I) At 25 mL/h PAN demonstrate an increase in fiber density (G), slight surface roughness (H), and a broader, more defined Taylor cone (I). (J–L) At 50 mL/h PAN display thicker, denser fibers (J), further surface roughness (K), and early signs of Taylor cone instability (L). (M–O) At 75 mL/h PAN show a well-defined fiber mat (M), rough surface texture (N), and a stable but larger Taylor cone (O). (P–R) At 100 mL/h PAN exhibit even denser fibers (P), rough surfaces (Q), and less stability in the Taylor cone (R). (S–U) at 125 mL/h PAN reveal a highly dense fiber mat (S), pronounced surface roughness (T), and increased Taylor cone instability, with polymer dripping from the jet (U). (V–X) At 150 mL/h PAN display thick, uneven fiber distribution (V), a highly rough surface (W), and significant instability in the Taylor cone (X), with frequent polymer dripping and inconsistent fiber production. The scale bar in (V) applies to all Overviews (40 μm), the scale bar in (W) applies to all Surface morphologies (400 nm), and the scale bar in (X) applies to all Liquid jets (1 mm).

The impact of increasing PAN core flow rates, from 1 to 150 mL/h, with a constant 10 mL/h sheath flow rate of tetrahydrofuran (THF) on fiber formation and Taylor cone stability was investigated. Figure 4 illustrates the transition from stable nanofiber production at lower flow rates to instability and wet fiber formation at higher flow rates. At low PAN flow rates (1 to 25 mL/h), the electrospinning process remains stable, producing fibers with smooth surfaces and consistent morphology. For instance, at 1 mL/h PAN (Figure 4A–C), the fiber mats are sparse and thin (A), and the surface morphology is smooth and uniform (B). The Taylor cone (C) is narrow and stable, ensuring uniform fiber formation. This trend continues at 10 mL/h PAN (Figure 4D–F) and 25 mL/h PAN (Figure 4G–I), with a slight increase in fiber density and the beginning of surface roughness. The Taylor cone remains stable at these flow rates, allowing for consistent fiber production. As the PAN flow rate increases to 50 mL/h (Figure 4J–L), the fibers become thicker and denser (J), with more pronounced roughness on the surface (K). The Taylor cone (L) starts to show good stability, and the overall process remains stable, leading to consistent fiber formation. The optimal flow rate for PAN when using THF as the sheath liquid was observed at 75 mL/h PAN (Figure 4M–O). At this flow rate, the fibers are well-defined (M), with rough surfaces (N) that are indicative of increased polymer content relative to solvent evaporation. The Taylor cone (O) at this point is stable, and larger than at lower flow rates, indicating that the system is operating near its maximum stable limit. The fiber morphology at this flow rate is consistent, making 75 mL/h the most favorable condition for high-throughput, stable nanofiber production with THF as the sheath. Beyond 75 mL/h, significant changes in both fiber morphology and jet stability are observed. At 100 mL/h PAN (Figure 4P–R), the fibers become even denser (P), with rougher surfaces (Q), and the Taylor cone (R) shows noticeable instability, leading to some inconsistency in fiber diameters. However, the electrospinning process still manages to produce fibers without major issues. At 125 mL/h PAN (Figure 4S–U) and 150 mL/h PAN (Figure 4V–X), the process becomes highly unstable. The fibers produced are denser and thicker (S,V), but their surface morphology becomes smoother (T,W), likely due to the excessive amount of polymer compared to solvent evaporation. This change in surface morphology suggests that the increase in solvent content relative to polymer leads to a smoother fiber appearance. However, the Taylor cone (U,X) at these flow rates exhibits significant instability, with frequent polymer dripping and the formation of secondary and tertiary jets. These small, side jets reduce the stability of the main jet, leading to inconsistent fiber diameters and wet fiber formation. The presence of these secondary jets indicates that the system is no longer capable of maintaining a single, stable Taylor cone at these high flow rates.

The investigation into varying the flow rates of the sheath liquid while keeping the core PAN flow rate constant is crucial for understanding the stability of the electrospinning process and fiber morphology. Figure S1 presents the effect of varying chloroform (CHCl_3_) flow rates with a constant 5 mL/h PAN core flow rate. At low chloroform flow rates (0.1 mL/h to 1 mL/h), the Taylor cone remained stable, producing fibers with smooth surfaces and consistent morphology. For example, at 0.1 mL/h CHCl_3_ (Figure S1A–C), the fibers exhibited typical PAN morphology, as the low chloroform content did not significantly affect the process. However, as the chloroform flow rate increased beyond 1 mL/h (Figure S1F–I), the Taylor cone began to elongate, indicating the onset of instability. Once the flow rate reached 1.5 mL/h and above (Figure S1J–L), the process became unstable, with visible polymer dripping and wet fiber formation. The destabilization is likely due to the increasing solvent content, which overwhelms the PAN solution, reducing the electrospinning efficiency and leading to polymer solution dripping onto the fiber mat. Figure S2 investigates the effect of varying diethyl ether flow rates while maintaining a constant 40 mL/h PAN core flow rate. At lower ether flow rates (0.1 mL/h to 5 mL/h), the Taylor cone remains stable, and the fiber mats are well-formed, with consistent surface morphology. For example, at 0.1 mL/h ether (Figure S2A–C), the fibers are thin and smooth, while at 5 mL/h ether (Figure S2G–I), fiber density increases slightly, and surface roughness begins to appear. However, as the ether flow rate increases beyond 5 mL/h, solvent accumulation on the aluminum collection surface was observed, indicating that the process had reached its stability limit. Above 5 mL/h, ether begins to disrupt the electrospinning process, leading to solvent drops collecting on the surface and affecting the fiber morphology. The highest stable flow rate for diethyl ether is determined to be 5 mL/h, beyond which the process destabilizes, and solvent dripping affects fiber formation. Figure S3 explores the effect of varying THF flow rates while holding the PAN core flow rate constant at 75 mL/h. At low THF flow rates (0.1 to 10 mL/h), the Taylor cone remains stable, and the fibers are consistent and well-formed. For instance, at 0.1 mL/h THF (Figure S3A–C), the fibers are thin and smooth, while at 10 mL/h THF (Figure S3J–L), fiber density increases, and surface roughness becomes more pronounced. However, as the THF flow rate increases beyond 10 mL/h, instability in the process becomes evident. At 20 mL/h and above, polymer dripping occurs, and the fibers exhibit significant irregularities, with solvent droplets wetting the fiber mat. The maximum stable flow rate for THF is determined to be 10 mL/h, beyond which the process destabilizes, leading to inconsistent fiber formation and solvent accumulation on the collection surface.

**Figure 5:**
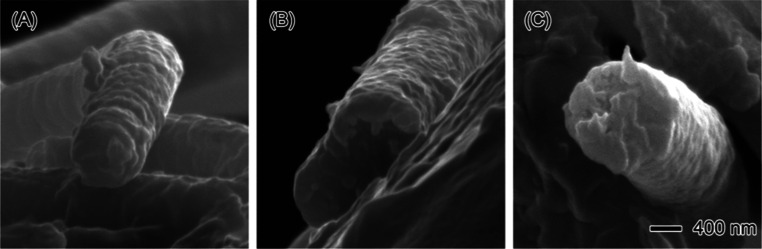
Cross-sectional SEM images of nanofibers produced under different flow rate conditions. (A) Shows a nanofiber produced with 5 mL/h PAN and 1 mL/h chloroform (CHCl_3_), exhibiting a smooth and wrinkled structure. (B) Presents a nanofiber formed with 40 mL/h PAN and 5 mL/h diethyl ether, showing a denser and more compact internal structure. (C) Illustrates a nanofiber produced with 75 mL/h PAN and 10 mL/h tetrahydrofuran (THF), displaying a rougher, and solid internal morphology. The scale bar in (C) applies to all images (400 nm).

The internal structure of PAN nanofibers plays a crucial role in their performance, particularly for applications where mechanical integrity and uniformity are essential.^61,62^ Typically, PAN nanofibers are known to exhibit a solid, nonporous internal morphology unless specifically engineered to have porosity, such as through the incorporation of other polymers or via post-treatment processes. In the case of this study, three different solvents—chloroform, diethyl ether, and THF—were used as sheath solutions during the electrospinning process to explore their potential influence on the nanofibers’ internal structure. As shown in Figure 5, cross-sectional SEM images of nanofibers produced using these solvents at their optimal flow rates reveal that the internal structure remains consistent across all samples. Figure 5A, which represents the nanofiber produced with 5 mL/h PAN and 1 mL/h chloroform, displays a solid, compact internal morphology with no visible signs of porosity. This indicates that the chloroform sheath does not significantly interact with the polymer jet in a way that would induce internal phase separation or pore formation. Similarly, Figure 5B, corresponding to the nanofiber produced with 40 mL/h PAN and 5 mL/h diethyl ether, shows a dense core with no evidence of porosity. Diethyl ether, like chloroform, primarily acts as a protective layer around the polymer jet, preventing premature evaporation of the core solvent, dimethylformamide (DMF), without altering the internal fiber structure. In Figure 5C, the nanofiber produced with 75 mL/h PAN and 10 mL/h THF presents a slightly rougher surface on the cross-section but still maintains a solid internal structure. Even at higher flow rates and with THF as the sheath solvent, the fiber’s internal morphology remains intact, with no indications of pore formation. This suggests that THF, like the other solvents, serves more as a stabilizing sheath for the core solution rather than influencing the fiber’s internal properties. These findings demonstrate that the choice of sheath solvent—whether chloroform, diethyl ether, or THF—does not significantly affect the internal structure of the PAN nanofibers. Instead, the solvents function primarily as protective barriers, preventing the DMF from evaporating prematurely during the electrospinning process. The fact that no internal porosity is observed in any of the fibers suggests that the solvents do not mix with the PAN polymer jet. Rather, their main role is to stabilize the electrospinning process and protect the integrity of the core solution.

**Figure 6:**
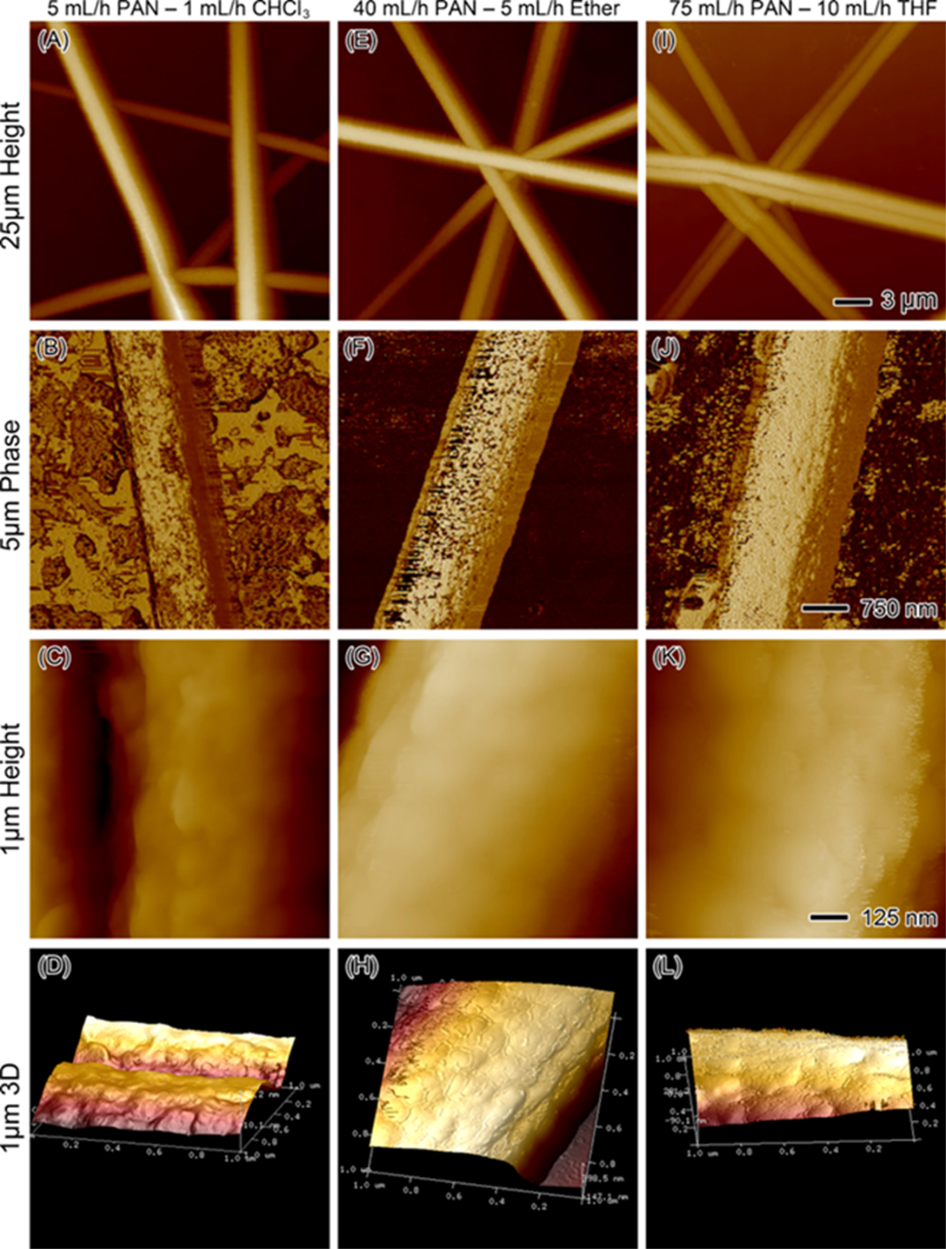
AFM images displaying the surface morphology of nanofibers produced with different sheath solvents. (A–D) Nanofiber produced with 5 mL/h PAN and 1 mL/h chloroform (CHCl_3_); (A) height image over a 25 μm scan, (B) phase image over a 5 μm scan, (C) height image over a 1 μm scan, and (D) 3D height representation over a 1 μm scan. (E–H) Nanofiber produced with 40 mL/h PAN and 5 mL/h diethyl ether; (E) height image over a 25 μm scan, (F) phase image over a 5 μm scan, (G) height image over a 1 μm scan, and (H) 3D height representation over a 1 μm scan. (I–L) Nanofiber produced with 75 mL/h PAN and 10 mL/h tetrahydrofuran (THF); (I) height image over a 25 μm scan, (J) phase image over a 5 μm scan, (K) height image over a 1 μm scan, and (L) 3D height representation over a 1 μm scan. The scale bars in the first row (A,E,I) apply to all height images with a 25 μm scan size (scale bar = 3 μm), the second row (B,F,J) applies to all phase images with a 5 μm scan size (scale bar = 750 nm), and the third row (C,G,K) applies to all height images with a 1 μm scan size (scale bar = 125 nm).

AFM was employed to investigate the surface structure of the nanofibers at a higher resolution than SEM. This technique provides valuable insights into the nanoscale surface morphology, offering more precise details about fiber surface characteristics such as roughness and texture. Figure 6 presents a comparative analysis of nanofibers produced using different solvent systems—chloroform, diethyl ether, and THF—at their respective optimal flow rates. In the case of the PAN/chloroform fibers (Figure 6A–D), the AFM height scan over a 25 μm area (Figure 6A) reveals that the fibers possess consistent diameters with a uniform, smooth surface. The phase image (Figure 6B) over a 5 μm scan highlights the homogeneity of the fiber surface, with no discernible features or surface roughness. A closer inspection using the 1 μm height scan (Figure 6C) further confirms the smoothness and consistency of the fiber surface. The 3D height representation (Figure 6D) shows a topographical view of the fiber, illustrating its smooth and solid structure. Overall, the AFM results indicate that the PAN/chloroform fibers exhibit a highly uniform morphology, with minimal surface texture or roughness. For the PAN/ether fibers (Figure 6E–H), the AFM scans similarly show consistent diameters and smooth surface characteristics. The 25 μm height scan (Figure 6E) demonstrates well-defined fibers with smooth surfaces, while the 5 μm phase scan (Figure 6F) reveals a uniform surface without any notable roughness or irregularities. The 1 μm height scan (Figure 6G) provides further confirmation of the smoothness of the fiber surface, and the 3D height representation (Figure 6H) highlights the smooth and regular topography of the fibers. These results suggest that the ether/PAN fibers closely resemble the PAN/chloroform fibers in terms of surface morphology and consistency. The PAN/THF fibers (Figure 6I–L) also exhibit similar surface characteristics to the other two solvent systems. The 25 μm height scan (Figure 6I) shows fibers with consistent diameters and smooth surfaces. The 5 μm phase image (Figure 6J) confirms the uniformity of the surface, with no visible roughness or texture. The 1 μm height scan (Figure 6K) reveals a smooth surface at a closer scale, and the 3D height representation (Figure 6L) provides a topographical view that supports the observation of smooth, uniform fibers. Across all three solvent systems—chloroform, ether, and THF—the AFM images consistently show that the nanofibers possess smooth, uniform surfaces with no significant differences in roughness or texture. The height and phase images, as well as the 3D representations, confirm that the surface morphology remains highly consistent regardless of the solvent used. This uniformity in surface characteristics across the different solvent/PAN flow rate combinations indicates that the choice of solvent does not significantly affect the nanoscale surface structure of the PAN fibers.

### PAN Nanofiber Production Quality and Quantity by LAUHS-ES

**Figure 7:**
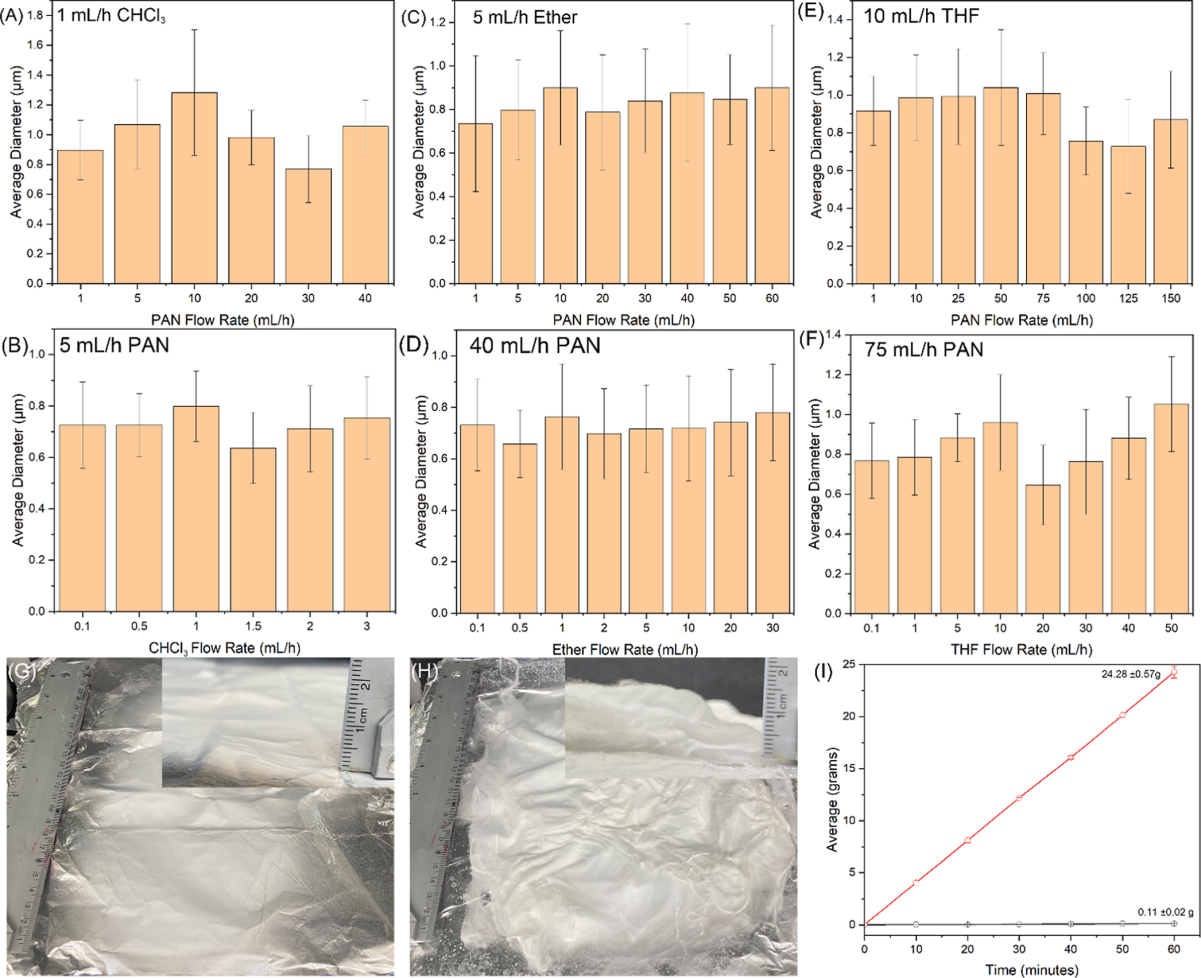
Average diameters of PAN nanofibers produced by LAUHS-ES under various flow rate conditions. (A) Shows the effect of varying PAN flow rates (1–40 mL/h) on fiber diameter with a constant chloroform (CHCl_3_) flow rate of 1 mL/h. (B) Illustrates the influence of varying CHCl_3_ flow rates (0.1–3 mL/h) on fiber diameter with a constant PAN flow rate of 5 mL/h. (C) Presents the effect of varying PAN flow rates (1–60 mL/h) on fiber diameter with a constant diethyl ether flow rate of 5 mL/h. (D) Shows the effect of varying ether flow rates (0.1–30 mL/h) on fiber diameter with a constant PAN flow rate of 40 mL/h. (E) Depicts the impact of varying PAN flow rates (1–150 mL/h) on fiber diameter with a constant THF flow rate of 10 mL/h. (F) Illustrates the effect of varying THF flow rates (0.1–50 mL/h) on fiber diameter with a constant PAN flow rate of 75 mL/h. Error bars represent the standard deviation of the measured diameters, reflecting variations in fiber uniformity under different flow rate conditions. Comparison of nanofiber production within 60 min using two different electrospinning methods. (G) Shows the fiber mat produced by conventional electrospinning, with a visibly thinner and less dense fiber layer (top right inset). (H) Displays the fiber mat produced by LAUHS-ES, demonstrating significantly higher fiber density and thickness (top right inset). (I) Presents a quantitative comparison of the mass of nanofibers produced over time by the two methods.

The diameters of the nanofibers produced by LAUHS-ES were measured from over 100 nanofibers in representative SEM images taken at 10,000× magnification. Figure 7 presents the diameter measurements for various combinations of PAN core flow rates and sheath liquid flow rates. Figure 7A,C,E show trials where the solvent sheath flow rate was held constant while varying the PAN flow rate, whereas Figure 7B,D,,F depict trials where the PAN core flow rate was held constant and the solvent sheath flow rate was varied. In Figure 7A, the fibers produced with 1 mL/h chloroform and varying PAN flow rates exhibited the largest average diameters, reaching 1.01 ± 0.159 μm. One possible explanation for this is the lower voltage applied during the chloroform trials, which results in a weaker electric field.^63^ The reduced force on the polymer solution as it emerges from the spinneret leads to thicker fibers because less stretching occurs during fiber formation. Additionally, the relatively low concentration of chloroform (only 1 mL/h) in these trials means that the PAN flow rate had a more pronounced effect on the resulting fiber diameters. As the PAN flow rate increased, the larger volume of polymer contributed to thicker fibers. In contrast, Figure 7B shows a narrower range of fiber diameters across varying chloroform flow rates, with diameters between 0.636 ± 0.138 and 0.800 ± 0.137 μm. This consistency is likely due to the small incremental changes in chloroform content, which had a relatively minor impact on the fiber diameter when compared to the influence of PAN. Figure 7C,D illustrate the effects of using diethyl ether as the sheath solvent, which produced the smallest fibers on average. Diethyl ether has the lowest boiling point among the three solvents tested, making it the most volatile. This higher volatility results in quicker solvent evaporation, leading to faster fiber solidification and smaller fiber diameters. The fibers produced with constant ether flow and varying PAN flow rates (Figure 7C) averaged 0.823 ± 0.054 μm, while the fibers produced with constant PAN and varying ether flow rates (Figure 7D) averaged 0.726 ± 0.036 μm. These fibers exhibited less variation in diameter compared to the chloroform trials, which suggests that ether promotes more uniform fiber formation due to its rapid evaporation. In Figure 7E, a trend of consistent fiber diameters is observed as the PAN flow rate increases from 1 to 75 mL/h while using 10 mL/h THF as the sheath solvent. However, as the PAN flow rate increases beyond 75 mL/h, the fiber diameters decrease significantly, with fibers produced at 100, 125, and 150 mL/h having average diameters of 0.756 ± 0.181, 0.728 ± 0.248, and 0.870 ± 0.257 μm, respectively. This decrease in fiber diameter at higher PAN flow rates corresponds to the transition from a stable to an unstable Taylor cone, as observed in the AFM and SEM images (Figure S3). Once the Taylor cone becomes unstable, the spinning process can no longer support the formation of larger fibers, leading to a reduction in fiber size. At very high flow rates the polymer jet travels rapidly from the spinneret to the collector. This reduces the available time for THF, acting as the sheath liquid, to evaporate and solidify the PAN fibers. As a result, the fibers remain wet longer and are subject to additional stretching forces during flight, leading to finer diameters. A similar trend is observed in Figure 7F, where the THF flow rate is varied while keeping the PAN flow rate constant at 75 mL/h. Fiber diameters remain relatively stable up to 10 mL/h THF, but a sharp drop in diameter occurs at 20 mL/h, which again corresponds to the onset of Taylor cone instability. Beyond this point, as the THF flow rate continues to increase, the fiber diameters show an upward trend, but this is accompanied by severe Taylor cone instability, resulting in wet fibers and inconsistent diameters.

The dramatic increase in fiber production rate represents one of the most significant achievements of this experiment. Using 75 mL/h PAN with 10 mL/h THF as the sheath solution in the LAUHS-ES setup, the production rate was compared to the conventional liquid-assisted electrospinning method with 1 mL/h PAN over a span of 60 min. Figure 7G depicts the fiber mat produced by the conventional electrospinning method at 1 mL/h PAN over the same time period. The fiber mat is considerably thinner, and the inset illustrates the lack of depth and density compared to the LAUHS-ES method. The conventional method yields a visibly less substantial mat, underscoring the limitations of conventional electrospinning in terms of production capacity. In contrast, Figure 7H highlights the fiber mat produced in 1 h using LAUHS-ES, which is visibly thicker and denser than that produced by the conventional method. The inset in Figure 7H shows the depth and compactness of the LAUHS-ES fiber mat, further emphasizing the efficiency of this method in producing high quantities of nanofibers. Figure 7I quantifies the comparison by plotting the mass of nanofibers produced over time, with data collected at 10 min intervals. The LAUHS-ES process produced an average mass of 24.28 ± 0.57 g in 60 min, whereas the conventional method yielded only 0.11 ± 0.02 g in the same time frame. This represents an increase in production yield by a factor of approximately 221 times when using LAUHS-ES. The significant boost in fiber output can be attributed to the optimized flow rate of the PAN solution in combination with the THF sheath in LAUHS-ES. The use of a higher polymer flow rate, combined with a stable sheath solvent system, allows for a continuous, stable Taylor cone, resulting in the rapid and consistent production of nanofibers. In contrast, the lower polymer flow rate of the conventional method, coupled with the lack of a stabilizing sheath solvent, leads to much slower fiber production and a reduced output over the same time period.

### Crystalline and Chemical Structures of PAN Nanofibers

**Figure 8:**
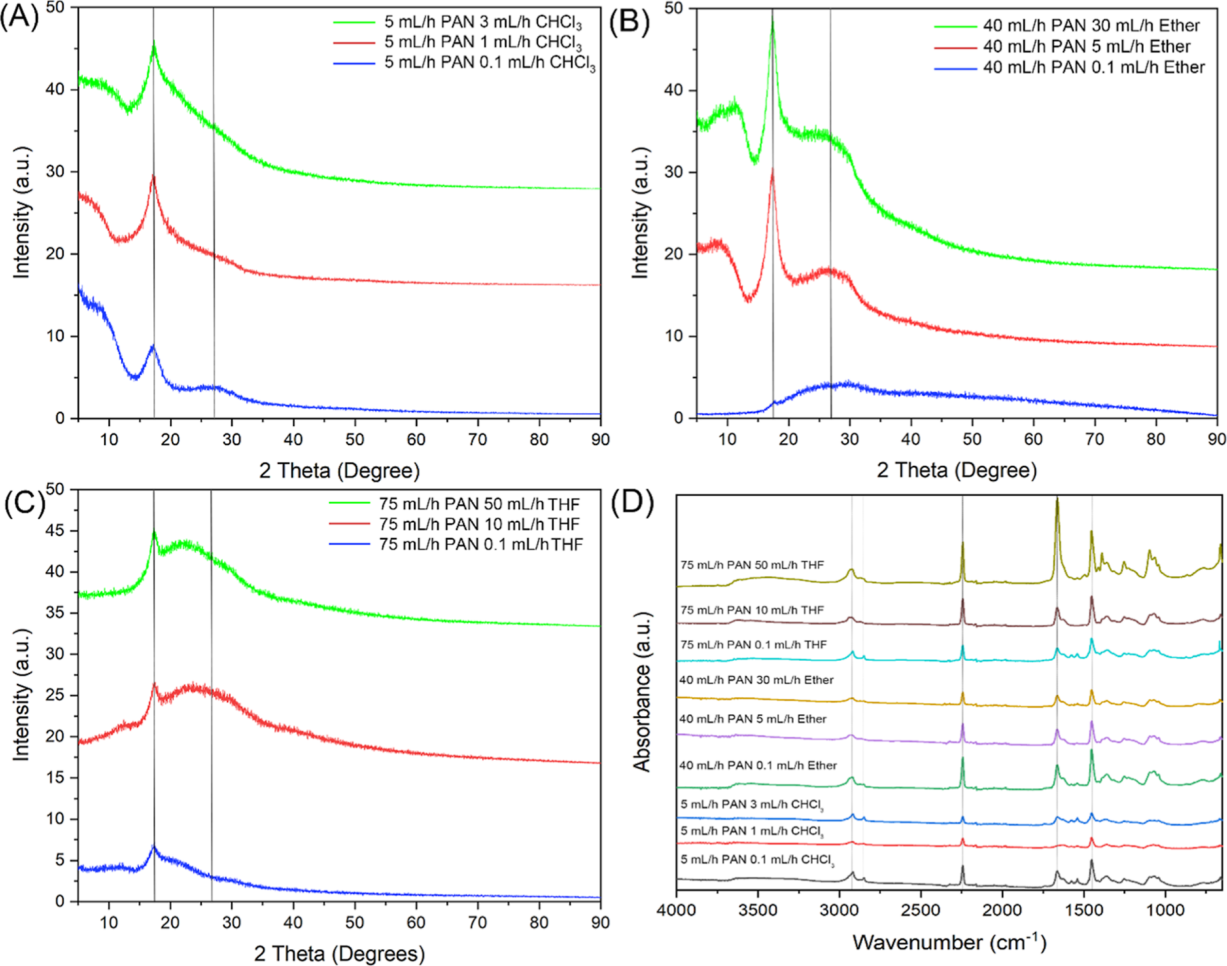
Comprehensive characterization techniques of the structure of the nanofibers. X-ray diffractograms of PAN nanofibers using different sheath liquids. (A) Shows the X-ray diffraction patterns for nanofibers produced with 5 mL/h PAN and varying chloroform (CHCl_3_). (B) Presents the X-ray diffraction patterns for nanofibers produced with 40 mL/h PAN and varying diethyl ether flow rates. (C) Displays the diffraction patterns for nanofibers produced with 75 mL/h PAN and varying THF flow rates. The vertical lines indicate the primary PAN diffraction peaks around 2θ = 17° and 29°, highlighting the semicrystalline structure of the nanofibers. (D) Infrared (IR) spectra of PAN nanofibers produced with varying core PAN and sheath solvent flow rates.

The crystallinity of the PAN nanofibers was assessed using XRD, with the results shown in Figure 8. PAN typically exhibits a broad diffraction peak around 2θ = 17.3°, corresponding to the (100) plane in a hexagonal structure.^64,65^ This characteristic peak is indicative of PAN’s semicrystalline nature, and the degree of crystallinity can be influenced by the processing conditions, such as the solvent flow rate used during electrospinning. As observed in Figure 8, at lower solvent flow rates, the PAN nanofibers show broader and less intense peaks, indicating a lower degree of crystallinity. For example, in Figure 8B, the nanofibers produced with 0.1 mL/h diethyl ether exhibit a broad peak at 16.8°, consistent with less crystalline, more amorphous PAN fibers. However, as the solvent flow rate increases, the peak becomes sharper and more intense, particularly at 30 and 50 mL/h ether flow rates. This sharpening of the peak suggests an increase in the crystallinity of the nanofibers as more solvent is introduced into the electrospinning process, promoting molecular alignment and polymer chain ordering. This trend is also evident in Figure 8A with increasing chloroform flow rates and in Figure 8C with increasing THF flow rates, where higher solvent flow rates similarly lead to sharper and more intense peaks around 17.3°. The increase in peak intensity and narrowing at higher solvent flow rates can be attributed to the cyclization of the PAN polymer chains, a process that enhances the crystalline regions within the fibers.^66^ Cyclization is a chemical transformation where the nitrile groups (–C≡N) in PAN interact, leading to the formation of a more ordered structure. The higher solvent content in the sheath allows for more uniform stretching and alignment of the polymer chains during electrospinning, which facilitates this transformation and results in fibers with greater crystallinity. This effect is most pronounced in Figure 8B, where the transition from a broad, low-intensity peak at 0.1 mL/h ether to a much sharper and more intense peak at 50 mL/h ether illustrates the significant impact that the solvent flow rate has on the crystallization of the PAN fibers. Similarly, Figure 8A,C show comparable trends, confirming that higher solvent flow rates, whether chloroform, ether, or THF, enhance the crystallinity of the nanofibers. This increase in crystallinity could improve the mechanical properties of the fibers, making them more suitable for applications that require higher strength and stability.

The effect of varying solvent flow rates on the chemical structure of PAN nanofibers was analyzed using ATR-FTIR spectroscopy. Figure 8D presents the IR spectra of the nanofibers produced with different solvent systems and flow rates. Across all spectra, the typical features of PAN are clearly evident, indicating that neither the polymer flow rate nor the choice of solvent significantly altered the chemical composition of the nanofibers. The characteristic PAN peaks are consistently observed in all samples. The broad peak around 2920 cm^–1^ corresponds to the asymmetric stretching of –CH_2_ groups, with a smaller peak at 2849 cm^–1^ representing the symmetric stretching of –CH_2_.^67^ The prominent peak at 2243 cm^–1^ is associated with the stretching vibration of the nitrile (–C≡N) group, which is a defining feature of PAN. Additionally, a connected peak at 1663 cm^–1^ also corresponds to nitrile stretching, further confirming the presence of the –C≡N groups in the nanofibers. The spectra also show a peak at 1453 cm^–1^, corresponding to the bending vibrations of –CH_2_ and –CH_3_.^68^ Further characteristic PAN peaks are observed at 1389 and 1360 cm^–1^, indicating CH_3_ bending, and at 1252 cm^–1^, corresponding to –CH bending. Additionally, a triple split peak is present at 1096, 1068, and 1041 cm^–1^, which are typically associated with C–O and C–N stretching vibrations.^69^ Importantly, despite the variations in both PAN and solvent flow rates, no new significant peaks appeared in the spectra. This indicates that the changes in flow rates or the choice of solvent had no detectable effect on the chemical structure of the nanofibers. The molecular structure of PAN remained unchanged across all conditions. This lack of variation in the chemical composition supports the findings from previous analyses (Figures 2–4 and S1–S3), where no significant differences in nanofiber morphology were observed apart from changes related to the wetting effects of higher solvent flow rates. These wetting effects were physical rather than chemical, and thus had no influence on the molecular structure of the fibers. The consistent appearance of characteristic PAN peaks across all samples suggests that the electrospinning process, regardless of solvent or flow rate variations, preserves the fundamental chemical integrity of PAN.

## Conclusions

In this study, we successfully developed and demonstrated a novel liquid-assisted ultrahigh-speed electrospinning (LAUHS-ES) technique that enables the production of high-quality PAN nanofibers at unprecedented throughput levels. By stabilizing the Taylor cone with a solvent sheath, the process achieves actual nanofiber production rates more than 200 times faster than conventional electrospinning methods, with zero compromises on nanofiber uniformity and morphology. Detailed analysis confirmed the structural integrity, smooth surface morphology, and consistent composition of the nanofibers across varying flow rates and solvent conditions. The use of three different solvent sheaths—chloroform, diethyl ether, and THF—provided insights into the effects of solvent choice on process stability and fiber morphology. THF at 75 mL/h PAN emerged as the optimal combination, producing nanofibers with stable morphology and consistent surface features. While increasing solvent flow rates generally led to higher instability and nonuniform fiber formation, the optimal solvent/PAN flow rate combinations maintained the structural and compositional integrity of the nanofibers, as evidenced by uniform fiber diameters and solid internal structures. The findings of this study suggest that LAUHS-ES holds significant potential for scaling up nanofiber production, addressing key limitations of traditional electrospinning methods. This technique offers an efficient, high-throughput solution for the commercial production of PAN nanofibers, which are critical precursors in applications such as carbon fiber manufacturing, filtration, and energy storage. Further optimization and adaptation of this technique could broaden its applicability to other polymer systems and nanofiber-based technologies, enabling more efficient and scalable production of advanced materials for various industries.

## Supporting Information Available

SEM images and liquid jet photographs illustrating the effect of varying sheath liquid flow rates while maintaining a constant PAN core flow rate (PDF)
