# Supplementary material for: Breaking through Electrospinning Limitations: Liquid-Assisted Ultrahigh-Speed Production of Polyacrylonitrile Nanofibers
Source: ACS Appl Eng Mater. 2024 Dec 3;2(12):2970–83. doi: 10.1021/acsaenm.4c00657 (PMC11686468; doi:10.1021/acsaenm.4c00657)
Supplement: Supplementary file 1 — em4c00657_si_001.pdf [file em4c00657_si_001.pdf]

Supporting Information

For

Breaking Through Electrospinning Limitations: Liquid-Assisted Ultrahigh-Speed Production of Polyacrylonitrile Nanofibers

John Schossig,[1] Qiangjun Hao,[1] Tyler Davide,[2] Adedayo Towolawi,[1] Cheng Zhang,[2] and Ping Lu[1,*]

[1] Department of Chemistry and Biochemistry, Rowan University, Glassboro, New Jersey 08028, United States

[2] Chemistry Department, Long Island University (Post), Brookville, NY 11548, United States

*Address correspondence to lup@rowan.edu (P. Lu).

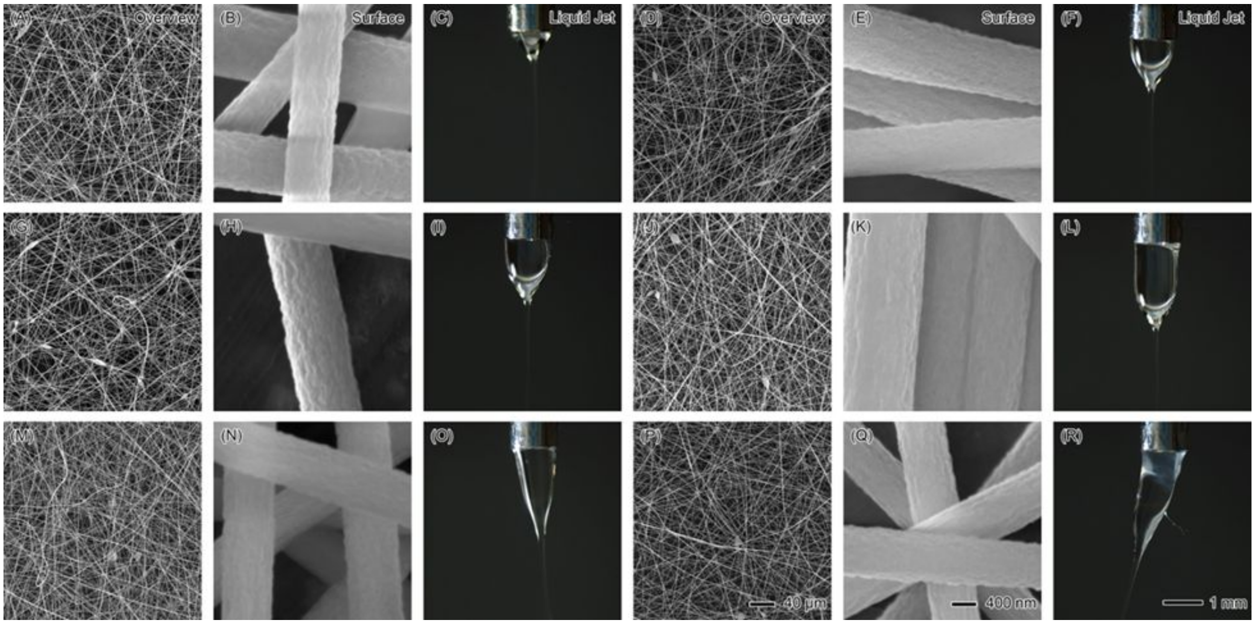

Figure S1. SEM images and liquid jet photographs illustrating the effect of varying sheath chloroform ($CHCl_3$) flow rates (0.1 mL/h to 3 mL/h) while maintaining a constant PAN core flow rate of 5 mL/h. (A-C): At 0.1 mL/h $CHCl_3$, the fibers appear sparse and thin (A), with smooth surfaces (B) and a stable, narrow Taylor cone (C). (D-F): At 0.5 mL/h $CHCl_3$, fiber density increases slightly (D), and the surface remains smooth (E), with minimal changes to the jet stability (F). (G-I): At 1 mL/h $CHCl_3$, fiber mats become denser (G), while the surface roughness remains minimal (H) and the Taylor cone maintains stability (I). (J-L): At 1.5 mL/h $CHCl_3$, the fibers are thicker and more interconnected (J), and the surface roughness increases slightly (K), though the Taylor cone (L) remains stable with minimal fluctuations. (M-O): At 2 mL/h $CHCl_3$, fiber production is denser (M), with increased surface texture (N), and the Taylor cone (O) begins to show early signs of instability. (P-R): At 3 mL/h $CHCl_3$, the fibers are densely packed (P), but the surface smoothness is maintained (Q), and the Taylor cone (R) shows noticeable instability, with some polymer dripping observed. The scale bar in (P) applies to all Overviews (40 µm), the scale bar in (Q) applies to all Surface morphologies (400 nm), and the scale bar in (R) applies to all Liquid jets (1 mm).

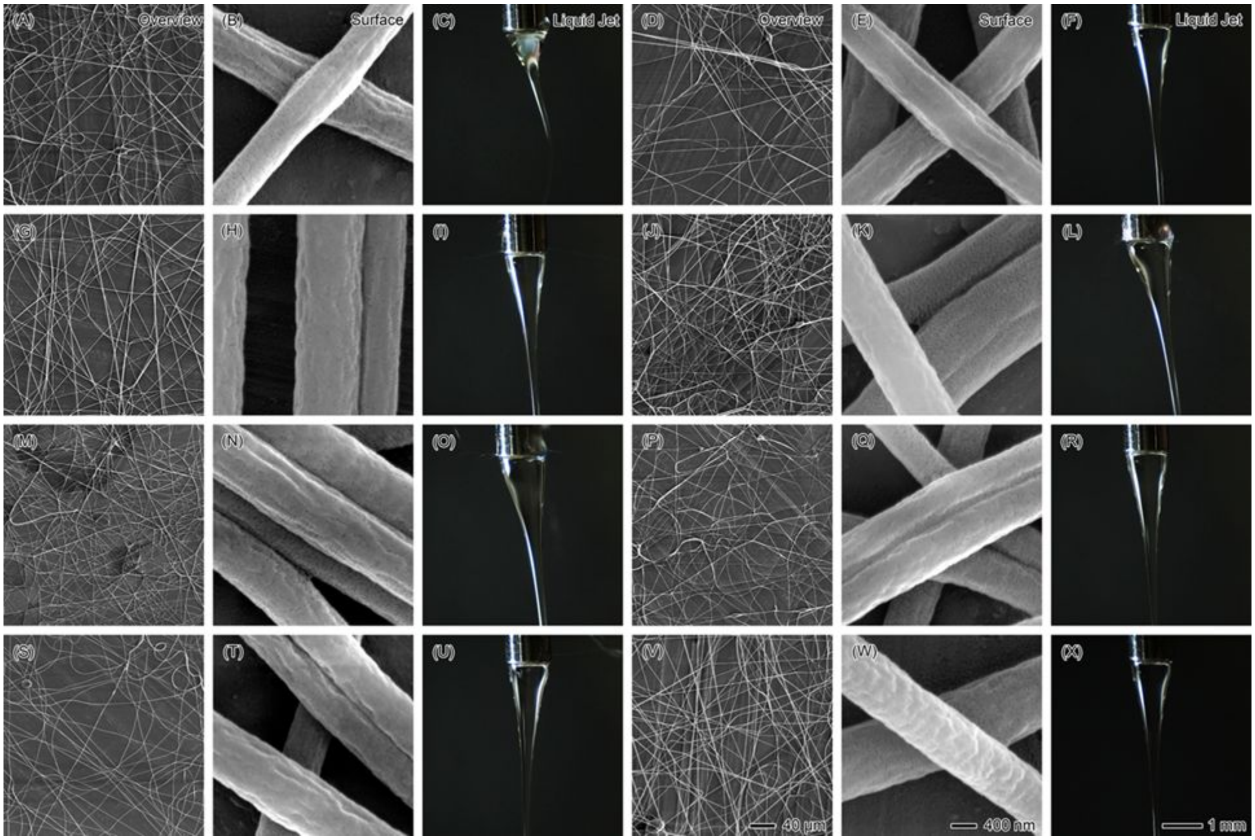

Figure S2. SEM images and liquid jet photographs illustrating the effects of varying ether flow rates (0.1 mL/h to 50 mL/h) while maintaining a constant PAN core flow rate of 40 mL/h. (A-C): At 0.1 mL/h ether, the fibers are sparsely distributed (A), with smooth surface morphology (B), and the Taylor cone (C) is narrow and stable, though small. (D-F): At 1 mL/h ether, the fiber density increases (D), with smooth surfaces (E), and the Taylor cone (F) remains stable but shows slight widening. (G-I): At 5 mL/h ether, the fiber mat becomes denser (G), the surface roughness increases slightly (H), and the Taylor cone (I) widens further but remains stable. (J-L): At 10 mL/h ether, fibers are more interconnected (J) and surface roughness is more pronounced (K), while the Taylor cone (L) starts to show early signs of instability, though it is still largely stable. (M-O): At 20 mL/h ether, the fibers become denser (M), with a rougher surface texture (N), and the Taylor cone (O) becomes visibly wider, indicating growing instability. (P-R): At 30 mL/h ether, the fibers appear thick and densely packed (P), with more pronounced roughness on the surface (Q), while the Taylor cone (R) becomes unstable, with some polymer dripping. (S-U): At 40 mL/h ether, the fiber mat remains dense (S), the surface exhibits significant roughness (T), and the Taylor cone (U) shows instability, leading to inconsistent fiber production. (V-X): At 50 mL/h ether, the fibers are densely packed (V), but the surface smoothness returns (W), likely due to excessive solvent,

and the Taylor cone (X) becomes highly unstable, resulting in polymer dripping and wet fibers. The scale bar in (V) applies to all Overview images (40 µm), the scale bar in (W) applies to all Surface morphologies (400 nm), and the scale bar in (X) applies to all Liquid jets (1 mm).

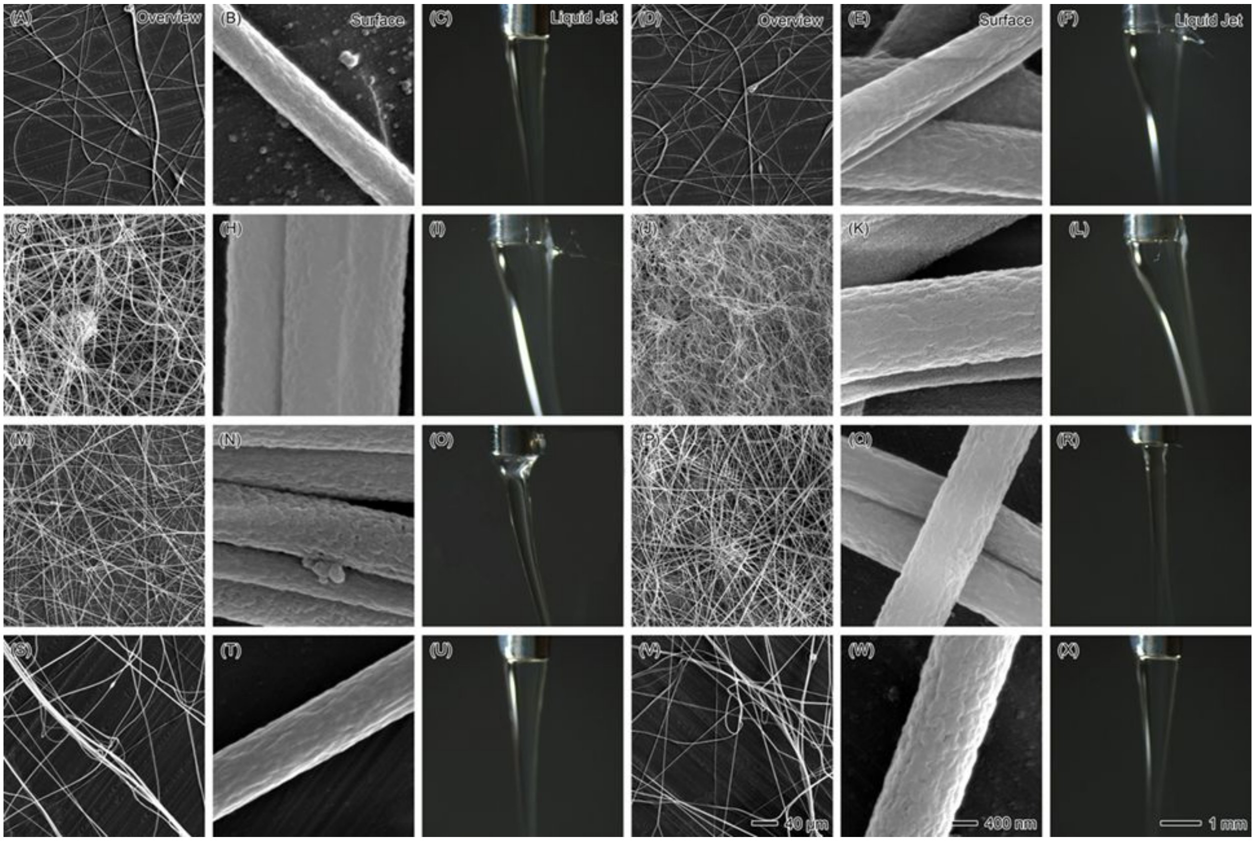

Figure S3. SEM images and liquid jet photographs illustrating the effect of varying THF flow rates (0.1 mL/h to 50 mL/h) while maintaining a constant PAN core flow rate of 75 mL/h. (A-C): At 0.1 mL/h THF, the fibers are sparse and thin (A), with smooth surface morphology (B), and the Taylor cone (C) remains narrow and stable. (D-F): At 1 mL/h THF, fiber density increases slightly (D), the surface remains smooth (E), and the Taylor cone (F) begins to widen but maintains stability. (G-I): At 5 mL/h THF, fiber mats become denser and more interconnected (G), the surface roughness increases (H), and the Taylor cone (I) widens further but remains stable. (J-L): At 10 mL/h THF, fibers are thicker and more densely packed (J), with further surface roughness (K), while the Taylor cone (L) starts to show early signs of instability, although the process remains relatively stable. (M-O): At 20 mL/h THF, the fiber mat density increases significantly (M), surface roughness is more pronounced (N), and the Taylor cone (O) widens, indicating growing

instability. (P-R): At 30 mL/h THF, fibers appear thick and densely packed (P), with rougher surfaces (Q), and the Taylor cone (R) becomes more unstable, with some polymer dripping. (S-U): At 40 mL/h THF, fibers are densely packed (S), surface texture is rough (T), and the Taylor cone (U) shows considerable instability, affecting fiber consistency. (V-X): At 50 mL/h THF, fibers are densely packed (V), but surface smoothness returns (W), likely due to excessive solvent content, while the Taylor cone (X) becomes highly unstable, leading to polymer dripping and inconsistent fiber formation. The scale bar in (V) applies to all Overview images (40 μm), the scale bar in (W) applies to all Surface morphologies (400 nm), and the scale bar in (X) applies to all Liquid jets (1 mm).
